# Insights into frankincense and myrrh research: A comprehensive analytical study of patterns and perspectives

**DOI:** 10.1016/j.heliyon.2024.e38102

**Published:** 2024-09-27

**Authors:** Siddig Ibrahim Abdelwahab, Manal Mohamed Elhassan Taha, Ahmed Ali Jerah, Abdullah Farasani, Saleh Mohammad Abdullah, Ieman A. Aljahdali, Omar Oraibi, Bassem Oraibi, Hassan Ahmad Alfaifi, Amal Hamdan Alzahrani, Yasir Osman Hassan Babiker

**Affiliations:** a Health Sciences Research Centre, Jazan University, Jazan, Saudi Arabia; b Department of Medical Laboratory Technology, Faculty of Applied Medical Sciences, Jazan University, Jazan, 45142, Saudi Arabia; c Department of Clinical laboratory sciences, Taif University, Taif, Saudi Arabia; d Department of Internal Medicine, Faculty of Medicine, Jazan, Jazan University, Saudi Arabia; e Pharmaceutical Care Administration (Jeddah Second Health Cluster), Ministry of Health, Jeddah, Saudi Arabia; f Department of Pharmacology and Toxicology, College of Pharmacy, King Abdulaziz University, Jeddah, Saudi Arabia; g Department of Surgery, College of Medicine, Jazan University, Jazan, 45142, Saudi Arabia

**Keywords:** Boswellia, Commiphora, Bibliometric analysis, Natural substances, Traditional medicine

## Abstract

**Objective:**

Frankincense (*Boswellia*) and Myrrh (*Commiphora*) are natural substances that have a long history of traditional use and potential therapeutic applications. This study aimed to provide comprehensive insights into the literature on Frankincense and Myrrh research (FMR) by examining patterns, perspectives, and research trends within the research landscape.

**Methods:**

This bibliometric study utilized MeSH-generated terms, followed the PRISMA guidelines, and analyzed English-based bibliographic data from original studies retrieved from the Scopus database. The VOSviewer and Bibliometrix applications were employed to analyze the CVS and BibTex data consisting of 955 records. This study focuses on publication trends, research topics, citation counts, research impacts, and collaboration dynamics.

**Results:**

The analysis revealed a steady increase in FMR, indicating growing interest in these substances. Egypt, the United States, and Saudi Arabia are the most prolific countries in terms of research output. FMR primarily focuses on chemical composition, pharmacological properties, and medicinal applications. Key research topics include identification and analysis of bioactive compounds, optimization of extraction techniques, and evaluation of their therapeutic potential. Surprisingly, the thematic map was overwhelmed by the niche, motor, basic, and emerging themes. Trending topics in FMR include “Myrrh oil”, “sesquiterpene”, “tapping”, “triterpenoids”, and “allergic contact dermatitis”. Collaboration networks highlight the involvement of diverse stakeholders, indicating the importance of multidisciplinary and international collaboration in advancing the field.

**Conclusions:**

These insights contribute to a better understanding of the research landscape of FMR, guiding future studies and facilitating the utilization of these natural substances for the benefit of society.

## 1 Introduction

Frankincense and Myrrh are ancient aromatic resins derived from trees in various species of the *Boswellia* (family Burseraceae) and *Commiphora* (family Burseraceae) genera, respectively. These natural substances have been highly valued throughout history for their diverse applications in medicine, spirituality, and perfumery [1,2]. Frankincense is obtained from various species of the *Boswellia* tree, primarily found in regions such as the Arabian Peninsula, India, and Africa [3]. At the same time, Myrrh is derived from various *Commiphora* species, primarily native to northeastern Africa and the Middle East [4].

The chemical compositions of Frankincense and Myrrh are complex and comprise a wide range of bioactive compounds. Frankincense is rich in boswellic acids, triterpenoids, essential oils, and other phytochemicals, whereas Myrrh contains sesquiterpenes, flavonoids, and other volatile constituents. These compounds contribute to the distinct aromatic profiles and potential health benefits [2,5,6].

Both Frankincense and Myrrh have traditionally been used for their medicinal properties. They are used as anti-inflammatory agents, analgesics, antimicrobials, and wound healers. In addition, these resins have been used in religious and spiritual practices, aromatherapy, and cosmetic products. Frankincense and Myrrh have a significant place in prophetic medicine. These aromatic resins were mentioned in ancient scriptures for their potential healing properties. They are used in various forms, including essential oils, incense, and topical applications for spiritual purification, respiratory health, skin conditions, and overall well-being, along with teaching prophetic traditions [1,3,[7], [8], [9]].

Bibliometric studies play a crucial role by providing quantitative insights into scholarly research. These studies offer valuable information on the intellectual structure of a field by identifying the key research areas, influential authors, and emerging trends. Using new analytical software, researchers can process vast amounts of data efficiently, uncover hidden connections, and visualize knowledge networks [10,11]. These quantitative methods complement narrative reviews by providing objective and evidence-based assessments of the research landscapes. They offer a comprehensive and systematic approach that allows researchers to make informed decisions, identify research gaps, and facilitate evidence-based policy making. Thus, bibliometric studies and analytical software significantly advance scientific knowledge and the understanding of intellectual structures [12,13].

Despite the long-standing historical use and potential therapeutic properties of frankincense and myrrh, a comprehensive bibliometric analysis of research conducted in Frankincense and Myrrh research (FMR) still needs to be improved. This study aims to fill this knowledge gap by analyzing research on frankincense and Myrrh. The analysis examined publication trends, research output, collaboration networks, and key contributors in the field. It employs various bibliometric indicators to gain insights into the growth and impact of research on FMR. The study also aimed to identify major research themes and subtopics, highlighting areas that require further exploration. These findings will benefit researchers, policymakers, and practitioners by providing a better understanding of the current research landscape and guiding future investigations.

## 2 Materials and methods

### 2.1 Choice of Scopus and search strategy

**Fig. 1:**
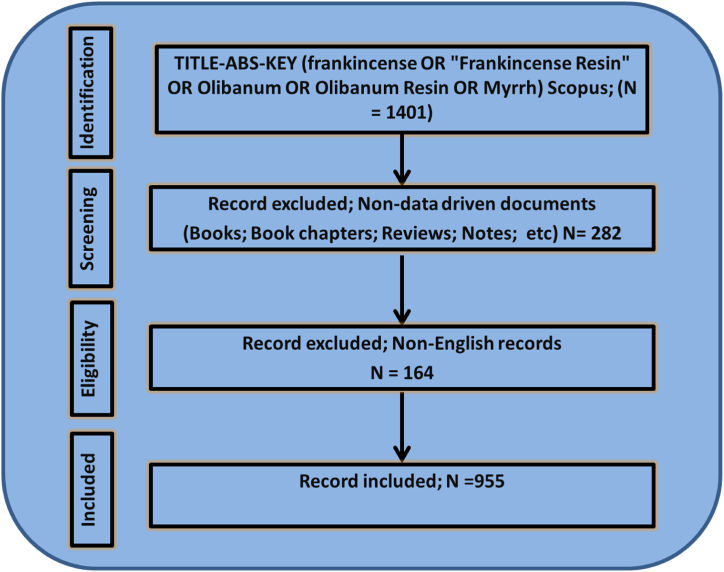
PRISMA guidelines for extracting the bibliographic data.

For several reasons, Scopus was preferred over other databases such as Web of Science (WOS), Google Scholar, and PubMed. Scopus is a comprehensive multidisciplinary database covering various scientific disciplines, including biomedical literature. It offers extensive coverage of scientific publications including journals, conference proceedings, and patents. Scopus also provides advanced bibliometric analysis tools and features, making it suitable for conducting comprehensive analyses [14]. The search strategy and study design followed the Preferred Reporting Items for Systematic Reviews and Meta-Analyses guidelines [15], as shown in Fig. 1. The search was performed in the Scopus database using the generated search terms and the inclusion criteria. The search terms were generated using the MeSH (Medical Subject Headings) database to ensure comprehensive coverage of the relevant keywords. The following search strings were used: TITLE-ABS-KEY (Frankincense OR "Frankincense Resin" OR Olibanum OR Olibanum Resin OR Myrrh) AND (LIMIT-TO (DOCTYPE, "ar")) AND (LIMIT-TO (PUBSTAGE, "final")) AND (LIMIT-TO (LANGUAGE, "English")). This search string was aimed at identifying articles with relevant keywords in the title, abstract, or section.

### 2.2 Inclusion criteria

The inclusion criteria for selecting documents for this analysis were as follows: (a) the document was written in English, (b) the document was finalized and published in a peer-reviewed journal, and (c) the document contained relevant information on Frankincense or Myrrh. These criteria ensured that only high-quality, peer-reviewed publications related to the topic of interest were included in the analysis. All other documents (reviews, book chapters, conference papers, notes, letters, books, editorials, short surveys, and conference reviews) are excluded. To focus on data-driven research articles, it is necessary to exclude these types of documents from bibliometric analysis. Excluding these publication types, the analysis prioritizes the original research studies that present empirical data and novel findings. Data-driven research articles provide a more robust foundation for bibliometric analysis, as they contribute directly to the advancement of knowledge and have a greater potential for impact. This exclusion helps ensure that the analysis focuses on rigorous scientific investigations, allowing for a more accurate assessment of research trends, patterns, and impacts within a specific field of study such as FMR. After applying the inclusion criteria, the final sample size of documents included in the analysis was 955. These documents formed the basis of the comprehensive bibliometric analysis conducted in this study.

### 2.3 Data analysis

VOSviewer, a software tool, was used to visualize and analyze the bibliometric networks. This helped to identify co-authorship networks, co-citation networks, and co-occurrence patterns within the literature. VOSviewer was used in this study to provide insights into collaboration patterns related to FMR [16]. Bibliometrix, an R package designed for bibliometric analysis, was used to extract key metrics and indicators from the dataset. It facilitates the calculation of descriptive statistics, collaboration analysis (e.g., co-authorship and institutional collaboration), and keyword analysis [17]. These analyses aided in identifying the publication trends, collaboration networks, and major research themes within the field. By leveraging the capabilities of VOSviewer and Bibliometrix, we conducted a comprehensive bibliometric analysis of FMR to uncover publication trends, collaboration networks, influential contributors, and major research themes. These tools facilitate a deeper understanding of existing literature and its bibliometric characteristics, enabling insights into Frankincense and Myrrh's scientific landscape. Total link strength (TLS) was used in the VOSviewer-produced maps to assess citations and collaboration.

## 3 Results

### 3.1 Main information about data

Table 1 presents the main information regarding the data of this bibliometric analysis conducted in Frankincense and Myrrh. This study covers a substantial timespan from 1838 to 2023 and includes 586 sources including journals, books, and other materials. A total of 955 documents were examined, highlighting the extensive body of research in this field. The study exhibited a steady annual growth rate of 2.24 %, demonstrating sustained interest over time. The average age of the analyzed documents was 9.38 years, indicating a mix of recent and older studies. Each document, on average, received 19.04 citations, highlighting the impact and recognition within the scholarly community. The analysis identified 9376 different Keywords Plus (ID) and 2575 author keywords (DE), demonstrating the diverse range of topics covered. This research involved 3541 authors, with 94 authors publishing single-authored documents. Collaboration was observed in 95 % of the documents, with an average of 4.93 co-authors per document. Furthermore, 28.96 % of the documents featured international co-authorship, showing global collaboration within the field.

**Table 1:** Main information.

Description	Results
Timespan	1838:2023
Sources	586
Documents	955
Annual Growth Rate %	2.24
Document Average Age	9.38
Average citations per document	19.04
Keywords Plus (ID)	9376
Author's Keywords (DE)	2575
Authors	3541
Authors of single-authored documents	94
Authors collaboration	
Single-authored document	95
Co-Authors per document	4.93
International co-authorships %	28.96

**Fig. 2:**
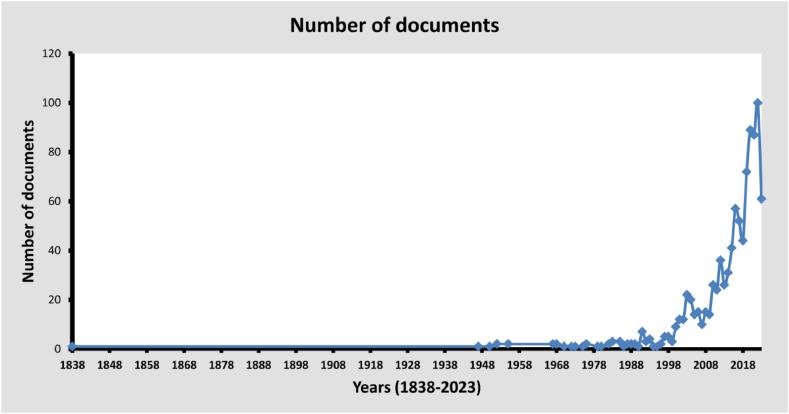
Annual publication trends of into frankincense and myrrh research (1838–2023). Y-axis: the number articles published. X-axis: the years since the first article published in the topic of this paper.

Despite its initiation in 1838, FMR has experienced a significant resurgence over the last 30 years, constituting 86.49 % of its overall growth. Over the past two decades, the research rate has increased by 65.76 %. Notably, the most substantial recovery was observed in the last five years, accounting for 42.62 % of the total. Fig. 2 illustrates research growth spanning more than a century, characterized by oscillations rather than a linear trajectory, as evidenced by a fourth-degree polynomial regression equation fit (R^2^ = 0.9265).

### 3.2 Hotspots and societal analysis

**Fig. 3:**
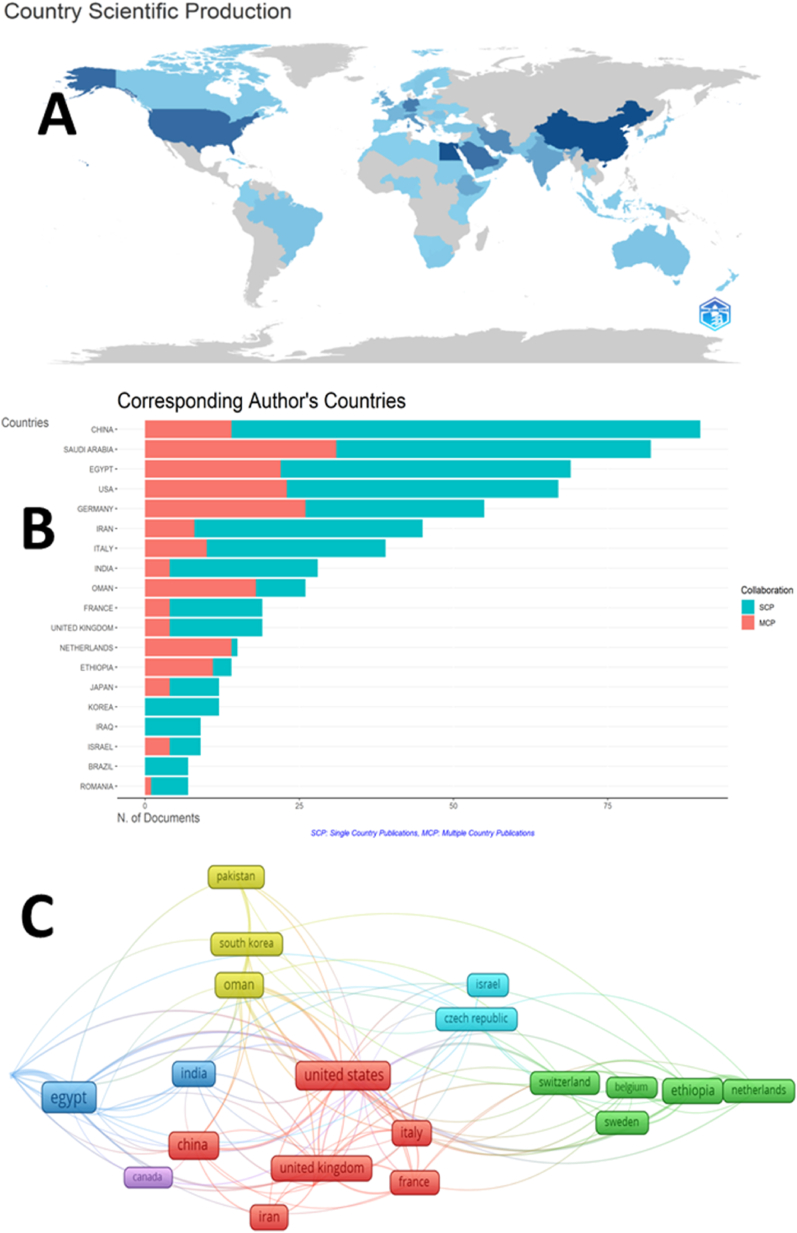
A: Global production. Countries with a dark blue color are the most productive. Countries outside the blue category have not made any contributions to research in this particular area. This figure was generated using the Bibliometrix application and the BibTex data file. B: Co-authorship mapping in terms of single-country publications (SCP; Aqua color), multiple-country publications (MCP, orange color), and the MCP ratio for each country. The MCP ratio indicates the extent of multiple-country collaborations in research publications, with higher ratios indicating a higher level of collaboration between countries. This figure was generated using the Bibliometrix application and the BibTex data file. C: Using VOSviewer software and applying a threshold of ten documents, the study examined research collaborations among 87 countries.

Egypt (10.91 %), the USA (8.82 %), Saudi Arabia (8.74 %), China (7.40 %), Germany (6.95 %), Iran (6.14 %), Italy (5.01 %), India (3.89 %), Ethiopia (3.52 %), and the UK (3.26 %) emerge as the most prolific countries in the FMR. Their collective output accounts for 71.15 % of global production in this field (Fig. 3A). Notably, several Middle Eastern countries, including Saudi Arabia, Iran, Oman, Pakistan, Iraq, Sudan, Yemen, and Kuwait, have contributed substantially, representing approximately one-third (32.5 %) of the world's research production. The ratio between single- and multiple-country publishing (Fig. 3B) serves as an indicator of international collaboration within a specific research domain or country's research output (Fig. 3B). Korea, Iraq, and Brazil exhibited a ratio of zero, indicating a lack of collaboration across multiple countries in their research publications. Conversely, the Netherlands and Ethiopia demonstrated high levels of international collaboration, with ratios of 0.933 and 0.786, respectively. Among the top five productive countries, Saudi Arabia and Germany exhibited the strongest engagement with other countries in the realm of FMR. Using VOSviewer software and applying a threshold of ten documents, this study examined research collaborations among 87 countries. The analysis revealed that 23 countries surpassed the threshold, forming six distinct clusters with combined link strengths of 351 and 115. Each cluster was visually differentiated using a unique color. Notably, the blue cluster encompasses Egypt, India, Saudi Arabia, and Japan, indicating higher collaboration among these countries. Fig. 3C highlights Egypt, the United States, Germany, Oman, and Saudi Arabia as leading countries in collaborative FMR. The corresponding total link strength (TLS) data for these countries are 69, 69, 64, 62, and 60, respectively. These findings demonstrate robust research collaborations and significant contributions of these countries to the fields of Frankincense and Myrrh. On one hand, the temporal progression of international collaboration in this field has seen the inclusion of certain countries in recent times, including Saudi Arabia, Iran, Oman, and China. Conversely, the United Kingdom, Canada, and Sweden initiated their research contributions before 2010.

**Fig. 4:**
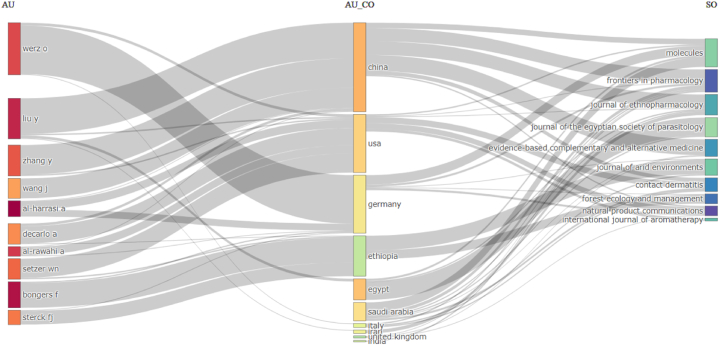
Three-field Sankay diagram. AU: author; AU_CO: country of the author; SO: source. The thickness of the lines connecting authors from different countries represents the number of papers they have co-authored. The thickness of the lines connecting countries and sources represents the number of papers from each country that have been published in each source. Each rectangle represents an author, country, or source. The size of the rectangle represents the importance of nodes in the network. This figure was generated using the Bibliometrix application and the BibTex data file.

King Saud University (Saudi Arabia), University of Nizwa (Oman), Wageningen University & Research (Netherlands), Al-Azhar University (Egypt), and the National Research Centre (Egypt), with 45, 29, 24, 24, and 21 documents, respectively, emerged as the leading institutions in terms of research productivity in the fields of Frankincense and Myrrh. Al-Harrasi, A. (Oman); Bongers, F. (Netherlands); Al-Rawahi, A. (Oman); Werz, O. (Germany); and Setzer, W.N. (USA) were the most prolific contributors. The dissemination of knowledge pertaining to these resins has been observed across various journals; however, the Journal of the Egyptian Society of Parasitology (N = 35), Molecules (N = 16), Journal of Arid Environments (N = 12), International Journal of Aromatherapy (N = 11), and Journal of Ethnopharmacology (N = 11) have accounted for a significant portion of these publications. Notably, these numbers represent absolute figures and do not consider the distribution of authors from different countries or the number of documents published in top-related journals within the field of research on these resins. The relationship between these factors can be explored further using a three-field Sankay diagram. Fig. 4 illustrates that Werz (Germany) attained the highest number of papers published in the top ten journals, thereby securing the leading position. Conversely, Saudi Arabia dropped to the sixth place, with the United States overtaking it as the second spot in terms of publication volume within this list, which comprised the most significant number of publications.

### 3.3 Citation mapping

Table 2 presents the top-cited documents in the field along with their respective citation counts and citation impact (CA). Among the top-cited documents, three stood out for their significance and impact. The most cited document, titled "Larvicidal effects of various essential oils against Aedes, Anopheles, and Culex larvae (Diptera, Culicidae)," was published in Parasitology Research in 2006. It has received a remarkable 431 citations, indicating its widespread recognition and influence in the field. With a citation impact of 23.94, this document has substantially contributed to research on the larvicidal effects of essential oils. The second most cited document, published in the International Journal of Aromatherapy in 2003, explores the "inhibition of 5-lipoxygenase by essential oils and other natural fragment extracts." It has amassed 303 citations, demonstrating its significant impact on our understanding of the inhibitory effects of essential oils. With a citation impact of 14.43, this document has been influential in aromatherapeutic research. The third most cited document, published in the Journal of Pharmacology and Experimental Therapeutics in 1997, focuses on the "inhibition of human leukocyte elastase by boswellic acids." This study received 211 citations, indicating its importance in the field of pharmacology. With a citation impact of 7.81, this document has contributed significantly to the knowledge on the inhibitory effects of boswellic acids on human leukocyte elastase. The citation average, also known as citation impact (CA), indicates the average influence of each document. The citation impact values for the top three documents are 23.94, 14.43, and 7.81, respectively. These values suggest that these documents received citations above average for publications in the field, signifying their significant impact and contribution to the research landscape.

**Table 2:** Top-cited documents.

Rank	Title	Journal	Year	Citation	CA
1	Larvicidal effects of various essential oils against Aedes, Anopheles, and Culex larvae (Diptera, Culicidae) [5]	Parasitology Research	2006	431	23.94
2	Inhibition of 5-lipoxygenase by essential oils and other natural fragment extracts [8]	International Journal of Aromatherapy	2003	303	14.43
3	Inhibition by boswellic acids of human leukocyte elastase [18]	Journal of Pharmacology and Experimental Therapeutics	1997	211	7.81
4	*Arbuscular mycorrhizal* fungi enhance photosynthesis, water use efficiency, and growth of Frankincense seedlings under pulsed water availability conditions [3]	Oecologia	2012	194	16.17
5	Acetyl-boswellic acids are novel catalytic inhibitors of human topoisomerases I and IIα [19]	Molecular Pharmacology	2000	181	7.54
6	Organic chemistry of embalming agents in Pharaonic and Graeco-Roman mummies [6]	Nature	2001	159	6.91
7	Antimicrobial activity of six essential oils against a group of human pathogens: A comparative study [20]	Pathogens	2019	155	31.00
8	A chemical investigation by headspace SPME and GC-MS of volatile and semi-volatile terpenes in various *Olibanum* samples [21]	Phytochemistry	2005	153	8.05
9	The phylogenetic history and biogeography of the Frankincense and Myrrh family (Burseraceae) based on nuclear and chloroplast sequence data [4]	Molecular Phylogenetics and Evolution	2005	148	7.79
10	Incensole acetate, an incense component, elicits psychoactivity by activating TRPV3 channels in the brain [22]	FASEB Journal	2008	141	8.81

The most common research topics identified in Table 2 encompass the larvicidal effects of essential oils, the inhibitory effects of essential oils and natural compounds, and the medicinal properties of Frankincense and Myrrh. Studies examining the larvicidal effects of essential oils against mosquito larvae have highlighted the potential of natural compounds in controlling mosquito populations. The inhibitory effects of essential oils on specific enzymes such as 5-lipoxygenase have been explored for therapeutic applications. Additionally, investigations into the medicinal properties of Frankincense and Myrrh have shed light on their phylogenetic history, geographic distribution, and potential therapeutic uses. Collectively, these findings contribute to the understanding and utilization of natural compounds in various research fields.

**Fig. 5:**
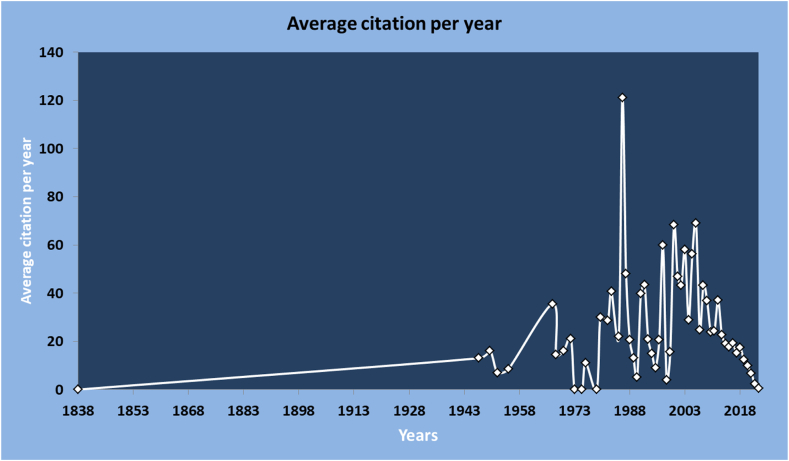
Average citation per year.

**Fig. 6:**
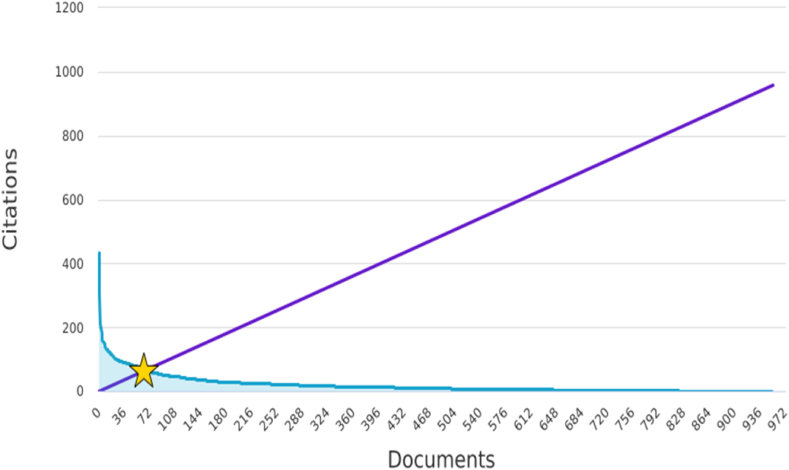
H-index.

Fig. 5 provides a summary of the citation averages for different years. The figure highlights the evolving trends in research impact and varying longevity of article citations across different publication years. The figure presents a comprehensive overview of the average citation counts per year for a wide range of publications from 1838 to 2023. Notable statistics include the highest average citation count of 121 in 1986 and several years, including 1838, 1973, 1975, 1979, and 2023, when the average citation count was 0. The year 2000 stands out with a significant average citation count of 68.33, indicating a substantial research impact during that period. The late 1980s and the early 1990s also exhibited relatively high average citation counts, suggesting a sustained interest and impact in the field. Conversely, 2019 to 2023 showed a declining trend in average citation counts, indicating reduced citation rates for articles published during those years. Overall, the table provides valuable insights into citation trends over time, showing variations in research impact and citation patterns across different publication years (see Fig. 6).

**Fig. 7:**
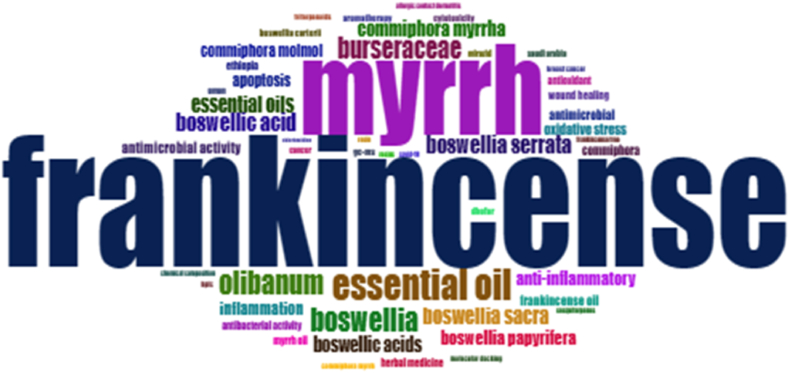
Keyword's cloud.

The h-index is a bibliometric indicator commonly used to evaluate the impact and productivity of researchers based on their publications and citations. While the h-index is typically applied to individual researchers, it can also be used to assess the impact of specific topics or fields of research, such as Frankincense and Myrrh. An h-index of 66 indicates that the researcher has published 66 papers, each of which received at least 66 citations (Fig. 7). The h-index provides a balanced measure of a researcher's influence, accounting for both the quantity and quality of their work. This reflects the researcher's ability to produce impactful papers that are widely cited.

### 3.4 Keyword co-occurrence and knowledge structure and distribution

The most frequent words in the given list were "Frankincense" (171 occurrences), "Myrrh" (118 occurrences), and "essential oil" (42 occurrences). Another term that appears frequently is "*Boswellia*" (32 occurrences), which refers to the genus of trees that produce Frankincense. The terms "boswellic acid" and "boswellic acids" (24 and 20 occurrences, respectively) were related to the bioactive compounds found in Frankincense. Other terms like "*olibanum*" (another name for Frankincense), "burseraceae" (the botanical family to which Frankincense and Myrrh belong), and "*Commiphora myrrha (Nees) Engl.*" (the scientific name for Myrrh) also appear in the list, highlighting their relevance to the topic. Among the associated concepts, there are terms like "anti-inflammatory," "antimicrobial activity," "oxidative stress," "apoptosis," and "antioxidant." These terms indicate the potential health benefits of Frankincense, Myrrh, and essential oils, which have been studied for their anti-inflammatory, antimicrobial, and antioxidant properties. Additionally, terms such as "wound healing," "inflammation," and "antibacterial activity" antibacterial activity suggest the potential applications of these natural substances in promoting healing and combating infections. Overall, the frequent occurrence of these terms reflects the significant interest in and research on the medicinal properties, traditional uses, and potential therapeutic applications of Frankincense, Myrrh, and essential oils. These frequent words are depicted in the word cloud in Fig. 7, which shows their significance and representation in the dataset. A word cloud is a visual representation of text data in which the size of each word corresponds to its frequency or importance within a given dataset. The frequency values were scaled to determine the relative size of each word in the word cloud. Generally, the more frequent a word, the larger it appears in the word cloud.

**Fig. 8:**
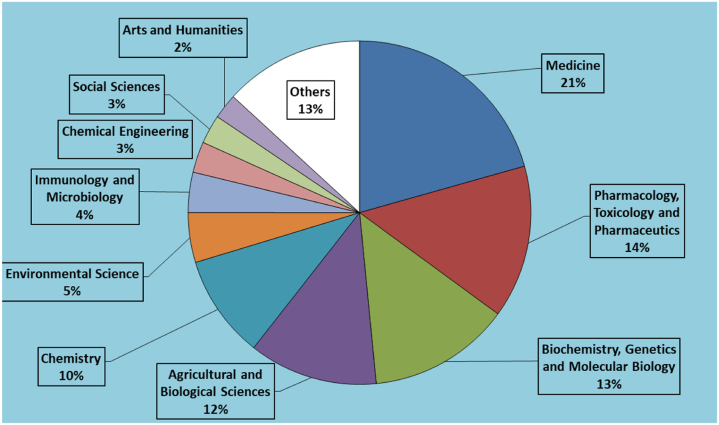
Subject area and information about the distribution of research publications across different subject areas. The field are Medicine (20.6 %), Pharmacology, Toxicology and Pharmaceutics (14.4 %), Biochemistry, Genetics and Molecular Biology (13.4 %), Agricultural and Biological Sciences (12.2 %), Chemistry (9.7 %), Environmental Science (4.7 %), Immunology and Microbiology (3.8 %), Chemical Engineering (2.9 %), Social Sciences (2.7 %), Arts and Humanities (2.4 %), and Other (Materials Science, Multidisciplinary, Physics and Astronomy, Earth and Planetary Sciences, Nursing, Engineering, Veterinary, Dentistry, Computer Science, Neuroscience, Energy, Health Professions, Economics, Econometrics and Finance, Mathematics, Business, Management and Accounting, and Psychology (13.2 %).

Fig. 8 provides information on the distribution of research publications across the different subject areas. Each subject area and the corresponding number of publications in a specific field are shown. The figure shows the relative prominence of the various subject areas based on the number of publications. Medicine had the highest number of publications (348), indicating significant research output and importance in the field. Pharmacology, Toxicology, and Pharmaceutics have 243 publications, signifying its focus on drug-related research and its impact on health. Biochemistry, Genetics, and Molecular Biology have 226 publications, highlighting the significance of molecular-level research in life sciences. Agricultural and Biological Sciences has 205 publications that emphasize research related to agriculture, ecosystems, and biodiversity. Chemistry has 164 publications with a focus on chemical research and its applications. Environmental Science has 79 publications, indicating research focused on environmental issues and sustainability. The figure also includes subject areas with relatively fewer publications, such as Economics, Econometrics, and Finance, with five publications; mathematics, with three publications; and Business, Management, and Accounting, with two publications, suggesting comparatively less research output in these areas. The information provided in the figure helps understand the distribution and relative emphasis of research across different subject areas, providing insights into the scientific landscape and areas of academic focus.

### 3.5 Thematic mapping

**Fig. 9:**
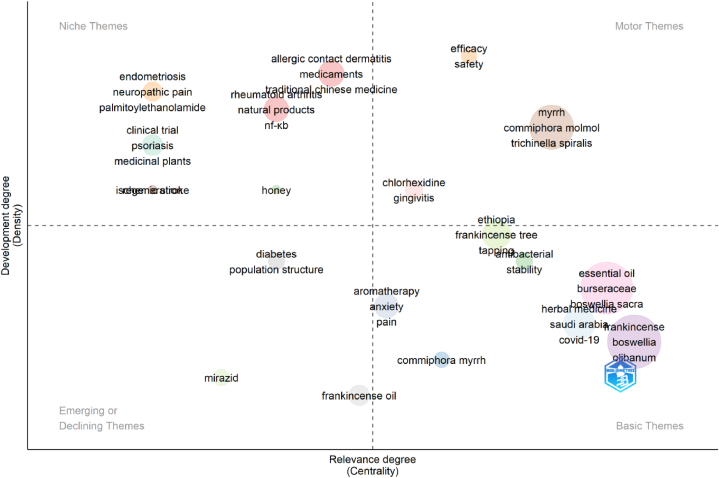
Thematic map. Thematic maps are divided into four quadrants based on centrality and density, which represent the importance and development of research topics. This figure was generated using the Bibliometrix application and the BibTex data file.

The thematic map of the FMR revealed various themes and their significance within the research landscape (Fig. 9 and Table 3). Centrality and density measures played crucial roles in determining the development and relevance of these themes. Centrality measures, such as Callon Centrality and Rank Centrality, assess the importance of a theme in the overall dataset. Higher centrality values indicate greater significance. For instance, "Frankincense" exhibited high centrality, suggesting its extensive study and relevance to research. Density measures such as callus density and rank density gauge the interconnectedness of a theme within its cluster. Higher density values imply strong associations with other related themes. "Myrrh" demonstrates a relatively high density, indicating its close connection to other themes within its cluster. The x- and y-axes placement of themes on the thematic map further helped determine their characteristics (Fig. 9). Themes located in the upper-right quadrant with higher centrality and density represent established and niche areas of research. "Rheumatoid arthritis," "ischemic stroke," and "endometriosis" fall into this category. Themes in the lower left quadrant, with lower centrality and density, may indicate emerging or less-studied topics. In this context, "essential oil" represents a basic theme, while "diabetes" may be an emerging or declining theme. Other themes, such as "honey," "efficacy," "clinical trial," and "regeneration," have varying centrality and density values, indicating their importance and relevance within the research landscape. By analyzing the thematic map and considering centrality, density, and the placement of the x- and y-axes, researchers gain insights into the development, relevance, and characteristics of different themes in FMR. This information assists researchers in identifying established areas, emerging trends, and potential connections, thereby enabling them to more effectively navigate the research landscape. Additional elaboration regarding the themes, their respective terms, and their classifications can be found in Table 3.

**Table 3:** Themes and their terms and classification.

Cluster label	Terms	N	Themes
Essential oil	Essential oil, Burseraceae, *B. sacra*, *C. myrrha*, Anti-inflammatory, Apoptosis, Antimicrobial activity, Antimicrobial, *Commiphora*, Wound healing, Antibacterial activity, Antioxidant, Myrrh oil, Cytotoxicity, Dhofar, Cancer, Oman, Molecular docking, Resins, Sesquiterpenes, *B. frereana*, Frankincense essential oil, Nanoemulsion, *Staphylococcus aureus*, Terpenoids, Traditional medicine, Biofilm, *Boswellia* spp., *C.* guidottii, Mouthwash, Scented Myrrh, Toothpaste, Triterpenes, Yemen	38	Basic
Frankincense	Frankincense, *Boswellia, Olibanum*, *B. serrata*, Boswellic acid, *B. papyrifera*, Inflammation, Oxidative stress, *B. carterii*, Breast cancer, HPLC, Triterpenoids, Chemical composition, Lipopolysaccharide, Non-timber forest products, Terpenes, Acetyl-11-keto-β-boswellic acid, *B. carteri*, GC–MS, Hippocampus, Network pharmacology, 5-lipoxygenase, AKBA, Pharmacokinetics	26	Basic
Myrrh	Myrrh, *C.* molmol, *Trichinella spiralis*, Albendazole, Chamomile flower, Coffee charcoal, Inflammatory bowel disease, Nanoparticles, Silver nanoparticles, Ulcerative colitis	10	Motor
Herbal medicine	Herbal medicine, Saudi Arabia, COVID-19, Herbs, Pregnancy, Diabetes mellitus	6	Basic
Rheumatoid arthritis	Rheumatoid arthritis, natural products, NF-ΚB, osteoarthritis, treatment	5	Niche
Ethiopia	Ethiopia, Frankincense tree, Tapping, Exclosure, Restoration	5	Motor
Allergic contact dermatitis	Allergic contact dermatitis, Medicaments, Traditional Chinese medicine, Herbal remedies	4	Niche
Diabetes	Diabetes, Population structure	3	Emerging or declining
Clinical trial	Clinical trial, Psoriasis, Medicinal plants	3	Niche
Aromatherapy	Aromatherapy, Anxiety, Pain	3	Basic
Endometriosis	Endometriosis, Neuropathic pain, Palmitoylethanolamide	3	Niche
Efficacy	Efficacy, Safety	2	Motor
Antibacterial	Antibacterial, Stability	2	Basic
Chlorhexidine	Chlorhexidine, Gingivitis	2	Motor
Ischemic stroke	Ischemic stroke	1	Niche
Honey	Honey	1	Niche
Regeneration	Regeneration	1	Niche
Frankincense oil	Frankincense oil	1	Emerging or declining
*C. myrrh*	*C. myrrh*	1	Basic
Mirazid	Mirazid	1	Emerging or declining

### 3.6 Thematic evolution

**Fig. 10:**
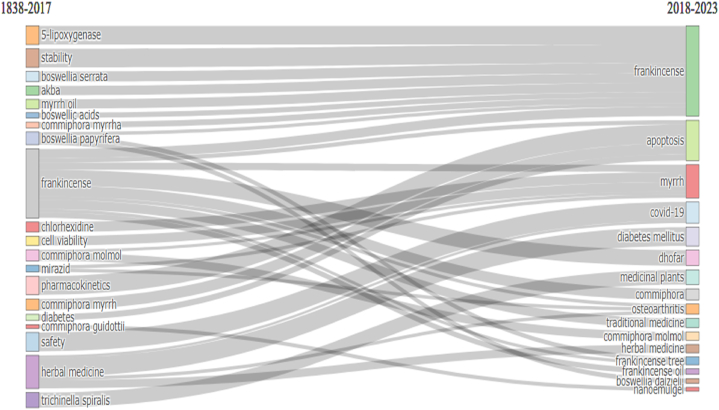
Thematic evolution. 2017 was a crucial point for the transformation of the main topics. This figure was generated using the Bibliometrix application and the BibTex data file.

Bibliometric analysis reveals the evolution of research interest over different periods. When comparing the two distinct time periods, it was possible to observe shifts in thematic focus. These changes reflect the dynamic nature of scientific inquiry and societal need. Fig. 10 depicts the thematic evolution of FMR before and after 2018. The figure shows the thematic evolution of the research from 1838 to 2017 and from 2018 to 2023. Several themes and their associations were presented, indicating a shift in research interest over time. 1838–2017 and 2018–2023. During the early period (1838–2017), topics such as 5-lipoxygenase, AKBA, *B. papyrifera*, *B. serrata*, boswellic acids, cell viability, chlorhexidine, *C. guidottii*, *C. molmol*, *C. myrrh*, diabetes, Frankincense, herbal medicine, medicaments, mirazid, myrrh oil, pharmacokinetics, safety, stability, and *Trichinella spiralis* were prominent. Research interest has shifted in the recent period from 2018 to 2023. The focus has expanded to include new topics, such as Frankincense, Frankincense tree, frankincense oil, *Commiphora*, COVID-19, Dhofar, apoptosis, osteoarthritis, traditional medicine, herbal medicine, diabetes mellitus, nanoemulsion, and medicinal plants. This evolution reflects a growing interest in exploring the therapeutic potential and applications of Frankincense and Myrrh as well as their interactions with various health-related aspects. The inclusion of COVID-19 as a research topic suggests that these natural substances should be examined during the pandemic. Advancements in technology and pharmaceutical formulations are evident through the emergence of topics, such as nanoemulsions. Overall, the thematic evolution in the bibliometric analysis demonstrates an expanding research landscape that encompasses a broader range of topics, and explores the potential benefits and applications of Frankincense, Myrrh, and related areas in different medical and scientific contexts.

### 3.7 Trend topics

The trend topics obtained using Bibliometrix revealed interesting insights into the research landscape across the different fields (Fig. 10). The analysis demonstrates the frequency and temporal distribution of various research areas, shedding light on the evolving scientific interest over time. For instance, the data highlight early publications on "medicaments" in the 1990s, indicating an initial exploration of this topic. On the other hand, "allergic contact dermatitis" shows a progressive increase in publications from 1996 to 2010, suggesting a growing focus on this area. Similarly, the trends for "sesquiterpene," "triterpenoids," "*B. carterii*," and "Myrrh oil" exhibit varying patterns of research output, indicating fluctuations and sustained interest in these respective subjects. The prominence of "GC-MS" analysis and the extensive study of "Burseraceae" further demonstrate the utilization and exploration of specific methodologies and plant families within the research community. Overall, these trends provide valuable insights into the temporal dynamics and popularity of research areas, thereby informing the scientific community of emerging and established fields of study.

## 4 Discussion

This study presents a pioneering effort to conduct a comprehensive analysis of Frankincense and Myrrh. By exploring existing literature, this study aims to provide insights into publication trends, research output, collaboration networks, and key contributors in this field. The findings will contribute to a deeper understanding of the scientific landscape surrounding Frankincense and Myrrh and provide a foundation for future research in this area.

Egypt's historical connection and geographical proximity to the Frankincense and Myrrh-producing regions [23] have contributed to its prolific research output on these substances. The country's rich cultural heritage and access to raw materials have fostered continued interest and investigation. Egypt's tradition of herbal medicine research and the presence of knowledgeable researchers have further enhanced its research capabilities [24]. The United States ranks second, owing to its robust scientific infrastructure, diverse population, and cultural ties to Frankincense and Myrrh. Saudi Arabia and Oman's direct access to Frankincense-producing regions, such as the Dhofar region [25], explains their research output. Ethiopia's rich biodiversity and traditional medicine practices have also contributed to its research on Myrrh [26]. Overall, these countries' historical, geographical, and cultural factors, along with their research traditions and resources, have positioned them as top contributors to the scientific understanding of Frankincense and Myrrh [27]. These results are consistent with those of previous studies, which show that Egypt and Saudi Arabia are among the countries with high research production in this field [23,28].

Research collaboration plays a vital role in enriching FMR by fostering knowledge exchange and resource sharing between researchers and institutions. Collaboration enables experts from different countries and disciplines to pool their expertise, perspectives, and resources, leading to a more comprehensive understanding of natural substances [29,30]. By working together, researchers can conduct large-scale studies, share data and methodologies, and explore diverse research approaches, ultimately advancing Frankincense and Myrrh's field. Creating bibliometric maps for research cooperation is an important measure of the strength of a research field. These maps visually represent the scientific output, collaborations, and impacts within the field of FMR. They help identify key contributors, influential institutions, and emerging trends, highlighting areas where further research cooperation can be fostered. Researchers and policymakers can identify potential gaps by analyzing bibliometric maps, establishing collaborative networks, effectively allocating resources, and promoting international partnerships. This collaborative approach enhances the overall quality and impact of FMR, leading to a deeper understanding of its properties, applications, and potential benefits in various fields, including medicine, pharmacology, and traditional practices [31].

The data showed fluctuations in citation counts over time, with some years showing higher citation averages. Fluctuations in citation count over time can be attributed to several factors. Changes in research trends and topic novelty can influence citation counts, with emerging areas attracting more citations [32]. Additionally, the volume of publications in a given year can impact citation potential, because a higher number of articles may lead to lower individual citation counts. The citation practices and behavior of researchers, along with variations in field-specific citation norms, can also affect citation counts. Furthermore, the quality and impact of research itself play a role, as high-quality studies tend to attract more citations. Finally, the time since publication also contributes, with newer articles requiring time to accumulate citations, while older ones may gradually receive fewer citations [33,34]. Our findings align with previous studies [35] on fluctuations in citation counts over time. Factors such as changes in research trends, volume of publications, citation practices, field-specific norms, research quality, and time since publication all contribute to the observed variations in citation averages [35,36]. These factors collectively influence citation patterns and the impact of articles published in different years.

The h-index is a bibliometric measure that evaluates the impact of a researcher's publications [37]. An h-index of 66 signifies that among the publications related to Frankincense and Myrrh, 66 articles each have garnered at least 66 citations. This indicates a substantial impact and recognition of FMR, suggesting that these topics have made significant contributions to the field. A high h-index reflects the influence and importance of studies exploring the medicinal properties, applications, and potential benefits of Frankincense and Myrrh, underscoring their relevance in disciplines, such as medicine, pharmacology, and biochemistry. It is important to note that the interpretation of the h-index can vary depending on the discipline and research area [38]. Generally, an h-index of 66 is considered to be quite high, indicating a significant impact and recognition. However, to make a meaningful comparison, examining h-index values from previous studies that specifically focused on Frankincense and Myrrh or related topics is necessary. No previous bibliometric studies have assessed FMR.

The word cloud summarizes the most prominent or frequently mentioned words in a given text or dataset. It helps identify key themes, topics, and recurring terms of the data. By visually representing the words, the word cloud allows for quick interpretation and analysis of textual information, highlighting the most significant words at a glance. It is often used as a visual aid in data exploration, text mining, and topic analysis [39]. The most frequent words in the given list were "Frankincense" (171 occurrences), "Myrrh" (118 occurrences), and "essential oil" (42 occurrences). The research focus on Frankincense outweighing Myrrh could be attributed to its longer historical and cultural association, which captures researchers' interests. Frankincense's century-old use in traditional medicine and religious contexts has sparked curiosity, leading to more studies. Additionally, the chemical composition of Frankincense, including bioactive compounds such as boswellic acids [18], offers a broader range of potential applications than Myrrh. The availability of research funding and resources may have also contributed to this discrepancy. However, research interests can shift over time and the current disparity may only partially reflect Myrrh's potential. As scientific understanding advances, interest in Myrrh and its applications may increase in the future.

The results of thematic map analysis provided valuable insights into the research landscape of Frankincense and Myrrh. Centrality measures such as Callon Centrality and Rank Centrality help determine the importance and significance of different themes [40]. The thematic map of the FMR shows a diverse range of topics and areas of interest within the field. It includes niche themes that focus on specific conditions or applications such as rheumatoid arthritis, allergic contact dermatitis, Endometriosis, Ischemic stroke, Honey, and Regeneration. These niche themes provide an in-depth exploration of particular aspects and attract researchers with specialized interests. On the other hand, Motor themes represent areas of active research and significant attention within the field. Examples include Myrrh, Ethiopia, and Chlorhexidine. These themes represent the driving forces of the research community, along with ongoing studies, advancements, and a substantial amount of published literature. The map also encompasses basic themes that cover the fundamental aspects of FMR. These themes, such as essential oils, Frankincense, Herbal medicine, Aromatherapy, Antibacterial, and *C. myrrh*, focus on the core properties, applications, and characteristics of the substances. This serves as the foundation for further exploration and understanding. In addition, the map features emerging or declining themes, such as Diabetes, Frankincense oil, and mirazid. The emerging themes indicate areas of growing interest and new research directions in the field. On the other hand, declining themes may represent topics that have been extensively researched in the past but are now experiencing a decrease in scientific interest or have reached a plateau. The combination of these diverse themes in the thematic map reflects the versatility and multidimensional nature of the FMR. It accommodates different research interests, allows for exploration of both established and emerging areas, and provides a comprehensive overview of the current landscape. Researchers can delve into specific niche areas, contribute to ongoing studies on motor themes, and explore foundational aspects covered by basic themes. A more detailed discussion of the major themes is presented in the following section.

The research theme was analyzed using Bibliometrix, which focuses on essential oils. The cluster label for this theme is "Essential oil," which encompasses a wide range of related terms and concepts. The analysis revealed 38 distinct terms associated with this cluster. This study highlights the various properties and activities associated with essential oils. For example, terms like "anti-inflammatory," "antimicrobial activity," "antioxidant," and "cytotoxicity" suggest that researchers are exploring the potential health benefits and therapeutic applications of essential oils. This includes their antimicrobial and anti-inflammatory properties as well as their potential role in wound healing and cancer treatment. The cluster also includes terms related to the chemical composition and analysis of essential oils. For instance, "sesquiterpenes," "terpenoids," and "triterpenes" triterpenes indicate that researchers are interested in the specific chemical compounds present in essential oils and their potential effects. The geographic context is also evident in this research theme. Terms such as "Dhofar," "Oman," "Yemen," and "Frankincense essential oil" suggest that researchers are investigating the traditional uses and production of essential oils in specific regions. Several studies have contributed to the understanding of essential oils and their potential applications. These studies include investigations into the cytogenotoxic and mutagenic effects of *C. myrrha* essential oil [41], the effects of massage with Frankincense and Myrrh oil in chronic low back pain [42], larvicidal effects of various essential oils against mosquito larvae [5], the biological activity of *B. serrata* essential oil [43], inhibition of 5-lipoxygenase by essential oils and natural extracts [8], the historical significance and modern applications of Frankincense oil [24], the potential of *B. rivae* for sustainable livelihood benefits [44], antimicrobial activity of essential oils against human pathogens, and an updated review of *C. molmol* and related species [23]. These articles collectively contribute to the multidisciplinary understanding of essential oils, encompassing their potential health benefits, therapeutic applications, chemical composition, extraction methods, and socioeconomic aspects. Overall, this thematic map analysis using Bibliometrix showcases diverse research interests and areas of focus within the research theme of essential oils. This demonstrates the multidisciplinary nature of the field, involving the aspects of chemistry, biology, medicine, and traditional knowledge.

The identified theme revolves around Frankincense (Table 3), specifically *Boswellia* spp. and their related compounds. The main areas of research within this theme include the chemical composition of frankincense, such as boswellic acid and terpenes, and their potential therapeutic applications. Inflammation and oxidative stress are significant topics, along with their implications for various health conditions such as breast cancer and hippocampal health [45]. Studies have also explored the pharmacokinetics and network pharmacology of frankincense and investigated its interactions and effects on the body [1,[46], [47], [48]]. Additionally, the theme encompasses the analysis of frankincense using techniques such as HPLC and GC-MS, highlighting the importance of the accurate identification and quantification of its constituents [21,48,49]. The inhibitory effects of frankincense on 5-lipoxygenase, an enzyme involved in inflammation, were explored [[50], [51], [52], [53], [54]]. Furthermore, the socioeconomic aspect of Frankincense is mentioned, particularly in relation to non-timber forest products [55]. Overall, this study provides a comprehensive understanding of the chemical, pharmacological, and socioeconomic aspects of frankincense and its potential applications.

Several studies have explored the inhibitory effects of frankincense on 5-lipoxygenase, an enzyme involved in inflammation. Ahmed et al. (2020) investigated the preventive effects of acetyl-11-keto-β-boswellic acid on testicular torsion and detorsion injury in rats by modulating the 5-LOX/LTB4 and p38-MAPK/JNK/Bax/Caspase-3 pathways. Alluri et al. (2020) demonstrated that the anti-inflammatory composition of *B. serrata* resin extract alleviates pain and protects cartilage in rats with osteoarthritis. Gilbert et al. (2020) provided structural and mechanistic insights into 5-lipoxygenase inhibition by natural products. Karlapudi et al. (2018) conducted a placebo-controlled double-blind study showing the clinical efficacy of a novel herbal formulation, including Frankincense, in relieving joint discomfort in individuals with knee osteoarthritis. Kim et al. (2020) investigated the anti-osteoarthritic effects of herbal compositions on human articular chondrocytes. Koeberle et al. (2018) identified triterpene acids from frankincense that inhibit 5-lipoxygenase. Shin et al. (2022) demonstrated that *B. serrata* extract prevented joint pain and cartilage degeneration in a rat model of osteoarthritis by inhibiting inflammatory responses. Stürner et al. (2020) found that lipid mediator profiles predict the response to therapy with oral frankincense extract in patients with MS. Yugandhar et al. (2018) conducted a placebo-controlled double-blind clinical study showing that an herbal composition containing extracts of *B. serrata* gum resin alleviates symptoms of asthma [[50], [51], [52], [53], [54],[56], [57], [58], [59]]. Collectively, these studies provide evidence for the inhibitory effects of frankincense on 5-lipoxygenase and its potential implications in inflammation-related conditions.

The cluster labeled "Myrrh" encompasses terms such as Myrrh, *C. molmol*, *Trichinella spiralis*, albendazole, chamomile flower, coffee charcoal, inflammatory bowel disease, nanoparticles, silver nanoparticles, and ulcerative colitis. This cluster covered a broad range of topics related to Myrrh, including its traditional use and potential applications in different contexts. These include its role in addressing parasitic infections, such as *Trichinella spiralis*, the use of albendazole as an anthelmintic medication [60], the potential effects of Myrrh, chamomile flower and coffee charcoal on health [61,62], and the association between Myrrh and inflammatory bowel disease, specifically ulcerative colitis [62]. In addition, the cluster includes terms related to nanoparticles, particularly AgNPs, and their potential applications in various fields. Several studies have explored the use of nanotechnology in Myrrh research. In one study, chemically and biosynthesized AgNPs were found to inhibit the viability and infectivity of *Trichinella spiralis* larvae. Another research project focused on the green synthesis of gold-conjugated polyphenol nanoparticles using Myrrh extracts, investigating their characterization, molecular docking, and biological applications. Stabilized Myrrh-capped hydrocolloidal magnetite nanoparticles were synthesized and evaluated for potential applications. Additionally, magnetic nanoparticles capped with Myrrh were investigated for their effectiveness in remediating water polluted with petroleum crude oil. AgNPs synthesized using a green method was assessed for their antileishmanial effects. The preparation and characterization of solid lipid nanoparticles loaded with Frankincense and Myrrh oil were explored as potential drug delivery systems. AgNPs synthesized using *C. mukul* extract were evaluated for their anti-arthritic activity. Furthermore, a combination of Myrrh and silver nanoparticles demonstrated superior antimicrobial activity against *Porphyromonas gingivalis* compared to their individual use. These studies collectively highlight the diverse applications of nanotechnology in Myrrh research, including its impact on parasites, characterization, environmental remediation, therapeutic potential, and antimicrobial properties [28,[63], [64], [65], [66], [67], [68], [69]].

The research theme of herbal medicine in the thematic map analyzed in this study (Table 3), particularly in the context of COVID-19, was reflected in several articles analyzed using Bibliometrix. These studies have explored different aspects of the potential application of herbal medicines in combating SARS-CoV-2. Caliebe et al. (2021) investigated the binding of boswellic acids to functional proteins of the virus, whereas Chitre et al. (2023) conducted a clinical trial on an Ayurvedic polyherbal formulation. Fatima et al. (2022) provided insights into β-boswellic acid and glycyrrhizic acid as potential inhibitors, and Gomaa et al. (2021) explored *B. serrata* extract as a therapeutic agent against COVID-19 in the elderly. Guarino et al. (2022) examined the effects of Curcuma longa and *B. serrata* on diabetic macular edema, and Hawkins et al. (2022) studied the impact of aromatherapy blends in post-COVID-19 patients. Jamshidi et al. (2022) reviewed the effects of *Boswellia* species on viral infections, and Kadhim et al. (2021) analyzed the inhibition of SARS-CoV-2 reproduction using *B. carterii*. Pham et al. (2023) investigated a natural product called YSK-A, and Roy and Menon (2022) evaluated the bioactive compounds from *B. serrata* against SARS-CoV-2. These articles collectively contribute to understanding the potential role of herbal medicine and exploring its interactions with the virus, anti-inflammatory properties, and therapeutic benefits in COVID-19 treatment [[70], [71], [72], [73], [74], [75], [76], [77], [78], [79]].

The theme of endometriosis is related to frankincense research. Endometriosis is a gynecological condition characterized by the presence of endometrial tissue outside the uterus that leads to chronic pelvic pain. Cho et al. (2023) and D'Amico et al. (2022) explored the potential effects of frankincense on endometriosis, a gynecological disease characterized by inflammation, oxidative stress, and dysregulated apoptosis. Cho et al. conducted a study using *in vitro* and *in vivo* models, suggesting that Frankincense reduces ectopic endometrial adherence and development through the ER stress/p53-apoptosis and chemokine-migration/adhesion pathways. D'Amico et al. used a rat model of endometriosis and found that *B. serrata* gum resin extract (a type of Frankincense) reduced the size and volume of endometriotic lesions, while regulating apoptosis and oxidative stress markers. These studies highlight the potential of Frankincense as a treatment option for endometriosis. However, further research is needed to fully understand its mechanisms and efficacy [[80], [81], [82]].

Frankincense and Myrrh, two aromatic resins with historical significance, have been the subjects of research related to aromatherapy, anxiety, and pain management. Buckle (2015) discussed the use of aromatherapy for stress, Cullen (2023) explored the effect of aromatherapy on cancer-related pain at the end of life, and Li et al. (2023) presented a case report of aromatherapy in carcinoma patients with malignant fungating wounds. Okano et al. (2019) investigated the effects of frankincense essential oil on stress in rats, Özdemir et al. (2023) conducted a trial on back massage with Frankincense and Myrrh oil for back pain, and Reis et al. (2022) evaluated frankincense essential oil for cancer-related fatigue. Additionally, Saylam et al. (2021) studied inhaler aromatherapy for pain and anxiety in patients undergoing shock-wave lithotripsy. Collectively, these articles provide insights into the potential benefits of aromatherapy, including the use of Frankincense and Myrrh for managing anxiety and pain [[80], [81], [82], [83]]. However, further research is required to establish its efficacy, optimal application methods, and mechanisms of action.

The thematic map focuses on the "Ischemic stroke" cluster within FMR, exploring their potential effects and applications in addressing ischemic stroke. Researchers have investigated the mechanisms, dosages, and interactions with existing treatments, highlighting their neuroprotective and anti-inflammatory properties. Existing studies have investigated the clinical efficacy and mechanisms of action of Frankincense and Myrrh in ischemic stroke [[84], [85], [86], [87], [88]]. They found that Frankincense showed potential clinical benefits in the acute phase of ischemic stroke, and the combination of Frankincense and Myrrh demonstrated synergistic effects in the treatment of cerebrovascular diseases. The mechanisms of action involve regulation of inflammatory pathways, such as TLR4/NF-κB, and modulation of angiotensin signaling pathways. These findings suggest that Frankincense and Myrrh may have neuroprotective effects and could be considered complementary therapies for ischemic stroke. However, further research, including clinical trials, is needed to validate their efficacy and determine the optimal treatment approaches.

Bibliometric analysis reveals the evolution of research interest over different periods [89]. When comparing the two distinct time periods, it was possible to observe shifts in thematic focus. These changes reflect the dynamic nature of scientific inquiry and societal need. For example, this analysis may reveal a transition from traditional medicinal practices to the exploration of the potential of natural products for the treatment of diseases. In addition, the emergence of novel technologies and methodologies can drive new research directions. By examining thematic evolution, researchers can gain insights into the evolving scientific landscape and identify emerging areas of interest that contribute to the advancement of knowledge and address current challenges.

**Fig. 11:**
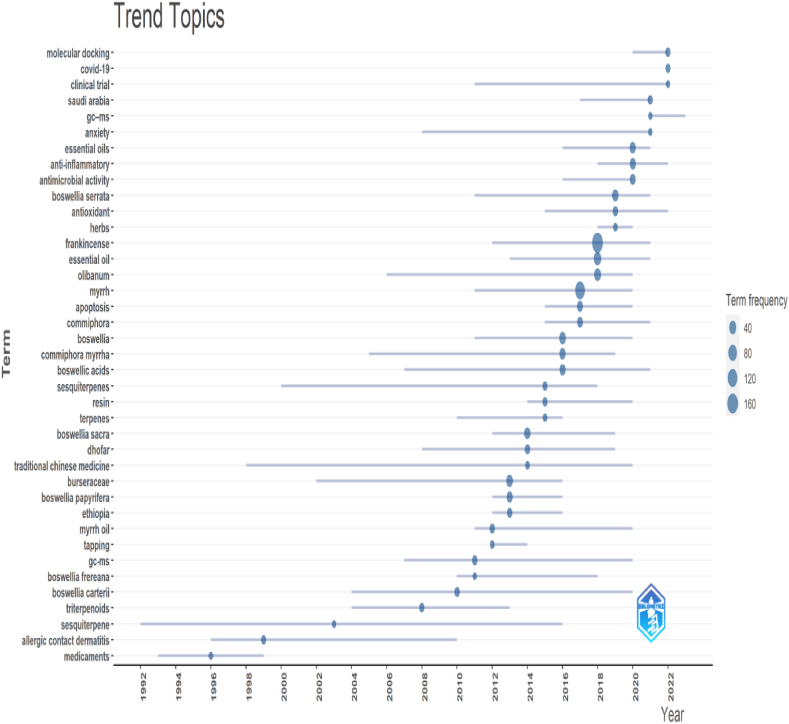
Trending topics. The graph depicts the research topic's time span, with horizontal lines indicating the duration and blue circles representing the frequency of the term. This figure was generated using Bibliometrix and BibTex data files.

Fig. 11 shows the trending topics. These topics are currently trending in FMR because of their significant contribution to the understanding of the chemical composition, therapeutic properties, and sustainable production of these natural substances. Gas chromatography-mass spectrometry (GC-MS) is a powerful analytical technique used to identify and quantify volatile compounds [49] present in Frankincense and Myrrh. They play a crucial role in characterizing the complex chemical profiles of these resins, identifying the key bioactive compounds, and assessing their quality and purity. Myrrh oil, derived from the resin of *Commiphora* species, has traditionally been used for its antimicrobial, anti-inflammatory, and wound healing properties [41]. Current research focuses on exploring the specific bioactive components in myrrh oil, their mechanisms of action, and their potential therapeutic applications in various fields [9,41,42]. *Boswellia frereana* is a frankincense tree known for its unique chemical composition and distinct aroma. Research on *B. frereana* has aimed to understand its specific bioactive compounds and their therapeutic potential. Sesquiterpenes, a class of aromatic compounds found abundantly in Frankincense and Myrrh, are of particular interest because of their pharmacological activity [90]. The Burseraceae family, to which Frankincense and Myrrh belong, is also an important area of study for understanding the phytochemical and therapeutic properties of these resins [4]. Finally, tapping, the sustainable extraction method for collecting resin from Frankincense and Myrrh trees, is a trending topic, as researchers explore environmentally friendly and economically viable approaches to ensure the long-term availability and conservation of these valuable resources [43]. The combined focus on GC-MS analysis, myrrh oil, *B. frereana*, sesquiterpenes, Burseraceae, and tapping demonstrates the multidisciplinary nature of FMR encompassing chemistry, pharmacology, sustainability, and conservation efforts.

## 5 Limitations

The bibliometric study described in the previous responses has limitations that should be acknowledged. By relying on English-based bibliographic data, language bias excludes non-English studies. The selection of Scopus as the sole database may require inclusion of relevant publications from other sources. Additionally, using citation count as the primary measure of research impact has drawbacks as it may need to fully capture the quality or real-world impact. This study lacks qualitative analysis, which can provide deeper insights. Furthermore, the findings may not be universally applicable because of the specific dataset and the time period considered. These limitations can help ensure a more comprehensive understanding of FMR.

## 6 Conclusions

In conclusion, the main findings of the discussed topics in FMR provide valuable insights into the chemical composition, therapeutic potential, and sustainability of these natural substances. GC–MS analysis has enhanced our understanding of the complex chemical profiles and bioactive compounds present in Frankincense and Myrrh. Research on Myrrh oil and *B. frereana* has highlighted their potential therapeutic applications in various fields, such as skincare and medicine. The significance of sesquiterpenes and the Burseraceae family in the pharmacological activities of Frankincense and Myrrh has been previously elucidated. Additionally, exploring sustainable extraction methods such as tapping is crucial for the long-term availability and conservation of valuable resources. The implications of these findings are vast, and can be extended to various stakeholders. It offers opportunities to develop novel pharmaceuticals, nutraceuticals, and skincare products from Frankincense and Myrrh. The sustainable practices recommended for resin extraction can help conserve natural resources and support the socioeconomic stability of communities that rely on their production. Furthermore, the findings emphasize the importance of conservation efforts and responsible sourcing in ensuring the long-term viability of Frankincense and Myrrh. Based on these findings, further research is recommended to explore the specific mechanisms of action, optimize extraction techniques, and conduct more extensive clinical studies to validate the therapeutic potential of Frankincense and Myrrh. This study provides valuable insights into the chemical composition, therapeutic potential, and sustainable practices of Frankincense and Myrrh. This highlights the complex chemical profiles, potential therapeutic applications, and importance of sustainable extraction methods. These findings offer opportunities for the development of novel products and emphasize the need for conservation efforts and responsible sourcing. Further research, collaboration, and clinical studies are recommended to optimize extraction techniques and validate their therapeutic potential. These recommendations will contribute to maximizing the potential benefits of Frankincense and Myrrh, while ensuring their conservation and sustainable utilization.

## Ethics approval and consent to participate

No human subjects were involved in this study; therefore, ethics approval was not required.

## Consent for publication

Not applicable.

## Availability of data and material

Data are attached as a supplementary material.

## Funding

The authors gratefully acknowledge the funding of the Deanship of Graduate Studies and Scientific Research, 10.13039/100009388Jazan University, Saudi Arabia, through Project Number: RG24-M014.

## CRediT authorship contribution statement

**Siddig Ibrahim Abdelwahab:** Writing – review & editing, Writing – original draft, Visualization, Validation, Supervision, Software, Resources, Project administration, Methodology, Investigation, Funding acquisition, Formal analysis, Data curation, Conceptualization. **Manal Mohamed Elhassan Taha:** Writing – review & editing, Writing – original draft, Formal analysis, Data curation, Conceptualization. **Ahmed Ali Jerah:** Writing – review & editing, Writing – original draft, Data curation, Conceptualization. **Abdullah Farasani:** Writing – review & editing, Writing – original draft, Data curation, Conceptualization. **Saleh Mohammad Abdullah:** Writing – review & editing, Writing – original draft, Funding acquisition, Conceptualization. **Ieman A. Aljahdali:** Writing – review & editing, Writing – original draft, Data curation, Conceptualization. **Omar Oraibi:** Writing – review & editing, Writing – original draft, Funding acquisition, Formal analysis. **Bassem Oraibi:** Writing – review & editing, Writing – original draft, Project administration, Methodology, Funding acquisition, Formal analysis, Conceptualization. **Hassan Ahmad Alfaifi:** Methodology, Investigation, Formal analysis, Data curation, Conceptualization. **Amal Hamdan Alzahrani:** Writing – review & editing, Writing – original draft, Visualization, Data curation, Conceptualization. **Yasir Osman Hassan Babiker:** Writing – review & editing, Writing – original draft, Validation, Supervision, Methodology, Investigation, Formal analysis, Data curation, Conceptualization.

## Declaration of competing interest

The authors declare that they have no known competing financial interests or personal relationships that could have appeared to influence the work reported in this paper.
